# Brain natriuretic peptide for prediction of mortality in patients with sepsis: a systematic review and meta-analysis

**DOI:** 10.1186/cc11331

**Published:** 2012-05-06

**Authors:** Fei Wang, Youping Wu, Lu Tang, Weimin Zhu, Feng Chen, Tao Xu, Lulong Bo, Jinbao Li, Xiaoming Deng

**Affiliations:** 1 Department of Anesthesiology and Critical Care, Changhai Hospital, Second Military Medical University, Shanghai, China; 2 Department of Nursing, Changhai Hospital, Second Military Medical University, Shanghai, China; 3 Department of Anesthesiology, General Hospital of Jinan Military Command, Jinan, China

## Abstract

**Introduction:**

Early identification of septic patients at high risk of dying remains a challenge. The prognostic role of brain natriuretic peptide (BNP) or N-terminal pro-B-type natriuretic peptide (NT-proBNP) in septic patients remains controversial. The purpose of this systematic review and meta-analysis was to investigate the value of elevated BNP or NT-proBNP in predicting mortality in septic patients.

**Methods:**

PubMed, Embase and the Cochrane Central Register of Controlled Trials were searched (up to February 18, 2011). Studies were included if they had prospectively collected data on all-cause mortality in adult septic patients with either plasma BNP or NT-proBNP measurement. Studies that failed to construct a 2 × 2 table of results were excluded. Two authors independently determined the validity of included studies and extracted data.

**Results:**

12 studies with a total of 1,865 patients were included. Elevated natriuretic peptides were significantly associated with increased risk of mortality (odds ratio (OR) 8.65, 95% confidence interval (CI) 4.94 to 15.13, *P *< 0.00001). The association was consistent for BNP (OR 10.44, 95% CI 4.99 to 21.58, *P *< 0.00001) and NT-proBNP (OR 6.62, 95% CI 2.68 to 16.34, *P *< 0.0001). The pooled sensitivity, specificity, positive likelihood ratio, and negative likelihood ratio were 79% (95% CI 75 to 83), 60% (95% CI 57 to 62), 2.27 (95% CI 1.83 to 2.81) and 0.32 (95% CI 0.22 to 0.46), respectively.

**Conclusions:**

Our results suggested that an elevated BNP or NT-proBNP level may prove to be a powerful predictor of mortality in septic patients. Future larger and more adequately powered prospective studies are warranted to clarify the assay standardization, the optimal cut-off, and the prognostic value of BNPs in conjunction with other biomarkers.

## Introduction

Sepsis is a leading cause of death in critically ill patients despite improvements in antimicrobial therapy and supportive care [1]. The septic response is an extremely complex chain of events involving inflammatory and anti-inflammatory processes, hormonal and cellular reactions, and circulatory abnormalities [2,3]. Early identification of patients at high risk of dying after intensive care unit (ICU) admission may help determine therapeutic interventions, such as changes in therapeutic protocols or further diagnostic procedures aiming at preventing shock and multiple organ failure with all their sequels that could have an impact on patients' outcome [4-6]. Therefore, there is a need for a fast simple and cost-effective method to enhance risk stratification in septic patients.

Brian natriuretic peptide (BNP) and its inactive cleavage product N-terminal fragment (NT-proBNP) were secreted into the blood in response to atrial or ventricular wall stretch [7], or myocardial ischemia [8] by cardiomyocytes. The half-life of BNP is approximately 20 minutes, and that of NT-proBNP is 1-2 hours [9]. The term BNPs will be used to represent either BNP or NT-proBNP throughout the rest of the paper unless otherwise stated.

BNPs have been found to be useful markers in the diagnosis, management and prognosis of patients with congestive heart failure [10]. In addition, BNPs are powerful predictors of death and major adverse cardiovascular events in patients with stable coronary disease [11], acute coronary syndromes [12] and pulmonary embolism, [13] and those who undergo noncardiac surgery [14]. Several prospective studies have been performed to investigate the potential role of BNPs in predicting mortality in septic patients, but they had limited numbers of patients, used different cut-off points and involved different clinical endpoints. In the present study, we made a systematic review and meta-analysis to evaluate the correlation between elevated levels of BNPs and death in septic patients.

## Materials and methods

This systematic review and meta-analysis was performed according to the guidelines of Meta-analysis of Observational Studies in Epidemiology [15].

### Study outcome

The aim of this meta-analysis was to see whether elevated BNPs could predict all-cause mortality in adult patients with sepsis.

### Search strategy and eligibility assessment

Pubmed, Embase and the Cochrane Central Register of Controlled Trials (up to February 18, 2011) were searched by using Exploded Medical Subject Headings and the appropriate corresponding keywords, "Brain natriuretic peptide", "B-type natriuretic peptide", "BNP", "pro-brain natriuretic peptide", "amino terminal pro-brain natriuretic peptide", "amino terminal pro-BNP", "N-terminal pro B-type natriuretic peptide", "NT-proBNP", "natriuretic peptide" AND "sepsis", "septicemia", "septicaemia", "septic". No language restrictions were applied. Additionally, the reference lists of the original studies and previous review articles were hand-searched to identify other potentially eligible studies.

Two authors independently determined the eligibility of all studies identified in initial research. Studies were included if they prospectively collected data on all-cause mortality in adult septic patients with either plasma BNP or NT-proBNP measurement. Studies where a 2 × 2 table of results could not be constructed were excluded. In case of disagreements, a third author was consulted. Agreement regarding study inclusion was assessed using the Cohen К statistic [16].

### Data extraction and quality assessment

Two authors independently extracted the following descriptive data from all eligible studies: study design, sample size, patient population, inclusion/exclusion criteria, diagnostic criteria for sepsis, severe sepsis and septic shock, follow-up period, completeness of follow-up, outcome assessment, marker evaluated (i.e., BNP or NT-proBNP), assay manufacturer, the optimal cut-off point, timing of BNP measurement, and the proportion of patients with an elevated BNP measurement. If data needed clarification or were not presented in the publication, the original authors were contacted by E-mail. Extracted data were entered into Microsoft Office Excel 2007 and checked by the third author. Any disagreement was solved by discussion.

Given that the eligible studies were of a prognostic nature, methodological and reporting quality was assessed according to the Quality Assessment of Diagnostic Accuracy Studies checklist (see Additional file 1) [17].

### Statistical analysis

Results were analyzed using Review Manager 5.1 (Cochrane Collaboration, Oxford, UK) and Meta-Disc 1.4 [18] (Clinical Biostatistics, Ramon y Cajal Hospital, Madrid, Spain). As individual studies used different cut-off points for defining elevated BNPs, Spearman's correlation coefficient between sensitivity and specificity was calculated to detect the presence of a threshold effect, where variations in sensitivity and specificity were related to differences in the cut-off point used to define an elevated BNPs level [19]. If there was no evidence of a threshold effect, then summary estimates, including odds ratio (OR), sensitivity, specificity, positive likelihood ratio, and negative likelihood ratio, were calculated using the random-effects model based on DerSimonian and Lair's meta-analytic statistical method [20]. In case a study provided multiple cut-off points for BNPs analysis, the point giving the maximum overall accuracy was chosen. In the case of analyses with empty cells, 0.5 was added to all cells to avoid computational errors. Publication bias was assessed by visually inspecting funnel plot. A p value of less than 0.05 was considered statistically significant.

Cochrane's Chi^2 ^test and I^2 ^test for heterogeneity were used to assess inter-study heterogeneity [21]. The Chi^2 ^test assesses whether observed differences in results are compatible with chance alone and the I^2 ^describes the percentage of the variability in effect estimates that is due to heterogeneity rather than sampling error. Statistically significant heterogeneity was considered present at Chi^2 ^*P *< 0.10 and I^2 ^> 50%. To explore the heterogeneity observed, sensitivity analyses were performed by sequential exclusion of each study. A prior subgroup analyses to explain significant heterogeneity were: 1) BNPs type (BNP versus NT-proBNP); and 2) blinding of BNPs measurement to the outcome (yes versus no). Single covariate random-effects meta-regression (inverse variance weights) was also used to explore sources of variation. Patient population (sepsis versus severe sepsis or septic shock), the underlying diseases (inclusion versus exclusion of preexisting conditions known to increase BNPs levels), optimal timing of BNPs measurement, and mortality were considered as variables.

## Results

**Figure 1:**
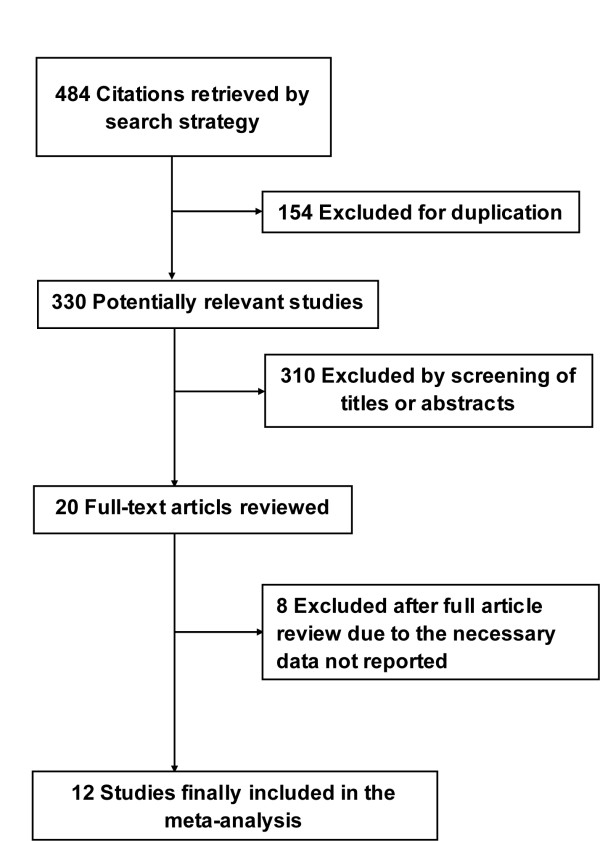
**Flow-chart of study selection**.

Our initial search yielded 484 citations, of which 464 were eliminated for various reasons based on the title and abstract. The full texts of the remaining 20 articles were scrutinized for further evaluation. Twelve studies [22-33] fulfilled our eligibility criteria and were finally included (Figure 1). The Cohen statistic К for agreement on study inclusion was 0.90.

### Characteristics of included studies

The included studies were published from 2004 to 2011 and all were prospective cohort studies (Table 1). Among them, seven studies [26-29,31-33] were conducted in Europe, three [24,25,30] in Asia, one [22] in North America, and one [23] in Australasia. Two studies [22,28] were multicenter studies and one study [23] reported both BNP and NT-proBNP. All studies but one [24] were published in English. The mean age of the patients varied between 55 and 69 years and the proportion of men ranged from 47 to 82%. The subject population varied across studies: three [22,25,26] included patients with sepsis, and nine [23,24,27-33] with severe sepsis or septic shock. The selected studies were performed in various departments, including emergency department, medical ICU, surgical ICU and general ICU. Nine studies [22,24,26,27,29-33] excluded patients with preexisting conditions known to increase BNPs levels. Follow-up periods differed across studies, including 28 days, ICU stay and hospital stay.

**Table 1:** Characteristics of included studies

Marker	Study	Country	Setting	Population	**NO**.	Male (%)	Age (year)	Outcome	Mortality (%)
NT-proBNP	Varpula 2007 [28]	Finland	24 ICUs	Severe sepsis or septic shock	254	69	59 ± 15	In-hospital mortality	26
	Mokart 2007 [29]	France	ICU	Cancer patients developing septic shock	51	63	56 (50 - 68)^†^	ICU mortality	51
	Roch 2005 [31]	France	General ICU	Septic shock	39	82.1	63 ± 12	ICU mortality	56
	Brueckmann 2005 [32]	Germany	Three departments in one hospital^‡^	Severe sepsis	57	74	55.0 ± 16.3	28-day mortality	28
	Sturgess 2010b* [23]	Australia	ICU	Septic shock	21	61.9	65 ± 17	In-hospital mortality	29
BNP	Perman 2011 [22]	USA	Emergency departments of 10 centers	Clinical evidence of sepsis	825	49	53.5 ± 19.6	A composite of events ^**§**^	6.6
	Zhao 2009 [24]	China	Surgical ICU	Severe sepsis or septic shock	102	53.9	**─**	28-day mortality	38.2
	Chen 2009 [25]	China	Emergency department	Sepsis	327	60.6	69.5 ± 13.4	28-day mortality	37.3
	Yucel 2008 [26]	Turkey	General ICU	Sepsis	40	**─**	**─**	28-day mortality	50
	Post 2008 [27]	Germany	ICU	Septic shock	93	55	65(53 - 73.5)^†^	30-day mortality	40.9
	Ueda 2006 [30]	Japan	Department of Emergency and Critical Care Medicine	Septic shock	22	77.3	62.5 ± 19.3	28-day mortality	54.5
	Charpentier 2004 [33]	France	Medical ICU	Severe sepsis or septic shock	34	47.1	56 ± 15.7	28-day mortality	29
	Sturgess 2010a* [23]	Australia	ICU	Septic shock	21	61.9	65 ± 17	In-hospital mortality	29

BNP = brain natriuretic peptide, NT-proBNP = N-terminal pro-B-type natriuretic peptide, ICU = intensive care unit. *The study reported both BNP (Sturgess 2010a) and NT-proBNP (Sturgess 2010b). ^† ^Median (25th - 75th percentiles). ^**¶ **^Dash indicates that information was not provided. ^**§ **^Events include in-hospital mortality, severe sepsis, or septic shock within 30 days following presentation. ^‡ ^Anesthesiology Department, Cardiosurgical Anesthesiology Department, and Internal Medicine Department.

### BNPs measurements

Among the included studies, BNP measurement was performed in eight [22-27,30,33] and NT-proBNP in five [23,28,29,31,32] (Table 2). In the BNP studies, four [22,23,25,27] used immunoﬂuorescence assay of Triage BNP test (Biosite Diagnostics, San Diego, CA), three [26,30,33] utilized immunoradiometric assay (Shionora-BNP), and one [24] did not report the method of BNP assay. In the NT-proBNP studies, four [23,28,29,31] used an electrochemiluminescence immunoassay performed on a Roche analyzer (Roche Diagnostics), and one study [32] utilized an enzyme immunoassay (Biozol). Among the included studies, receiver operating characteristic (ROC) curve was performed to retrospectively determine the optimal cut-off point with regard to 28-day mortality in seven studies [24-27,30,32,33], ICU mortality in two studies [29,31], in-hospital mortality in two study [23,28], and a composite outcome of in-hospital mortality, severe sepsis, or septic shock in one study [22]. Normal BNPs levels were defined as levels beneath or equal to the optimal cut-off points. The optimal cut-off points varied greatly across studies, from 32.1 pg/ml to 681.4 pg/ml for BNP, and from 400 pg/ml to 13,600 pg/ml for NT-proBNP. There were wide variations about the optimal timing of BNP measurement (six studies [22,24-26,28,31]: the day on admission; five studies [23,29,30,32,33]: day 2 after admission; one study [27]: day 5 after admission).

**Table 2:** NT-proBNP and BNP measurements

Marker	Study	Assay^†^	Optimal Timing	Cut-off Point (pg/ml)	Sensitivity/Specificity	AUC^¶^	Proportion of Elevated BNPs (%)
NT-proBNP	Varpula 2007 [28]	Roche, Elecsys 2010 analyzer	On admission	7090	58/66	0.631	40.6
	Mokart 2007 [29]	Roche, Elecsys 2010 analyzer	Day 2 after admission	6624	86/77	0.87	54.9
	Roch 2005 [31]	Roche, Elecsys 2010 analyzer	Within 24 hours after admission	13600	73/83	0.8	48.7
	Brueckmann 2005 [32]	Enzyme immunoassay (Biozol)	Day 2 after admission	11900	50/90	0.68	21.1
	Sturgess 2010b* [23]	Roche, Elecsys 2010 analyzer	Within 72 hours after admission	400	83/40	0.67	66.7
BNP	Perman 2011 [22]	Biosite Diagnostics, Triage	On admission	49	63/69	0.69	47.6
	Zhao 2009 [24]	Immunofluorescence assay^**‡**^	24 hours after admission	681.4	91.4/80.3	0.915	47.1
	Chen 2009 [25]	Biosite Diagnostics, Triage	Within 24 hours after admission	113	86/55	0.737	32.1
	Yucel 2008 [26]	Immunoradiometric assay, Shionoria	On admission	32.1	100/95	0.99	52.5
	Post 2008 [27]	Biosite Diagnostics, Triage	Day 5 after admission	121	76.3/52.7	0.648	59.1
	Ueda 2006 [30]	Immunoradiometric assay, Shionoria	Day 2 after admission	650	92/80	0.85	59.1
	Charpentier 2004 [33]	Immunoradiometric assay, Shionoria	Day 2 after admission	190	70/67	0.66	44.1
	Sturgess 2010a* [23]	Biosite Diagnostics, Triage	Within 72 hours after admission	254	83/60	0.76	52.4

BNP = brain natriuretic peptide, NT-proBNP = N-terminal pro-B-type natriuretic peptide. *The study reported both BNP (Sturgess 2010a) and NT-proBNP (Sturgess 2010b). ^**† **^Manufacture and kind of assay. ^**‡**^The manufacture was not reported. ¶ AUC indicates the area under the receiver operating characteristic curve.

### Study quality and publication bias

All the twelve included studies fulfilled the requirements of a representative spectrum of patients, and clearly described selection criteria, outcome verification in the whole cohort, equal outcome evaluation regardless of the BNPs results, sufficient description of BNPs measurement for replication and availability of clinical data. Four studies [22,28,30,33] stated that the BNPs results were interpreted without knowledge of outcome assessment. Because the target condition was mortality, outcome assessment was unlikely to be influenced by knowledge of the BNPs results. No patient was lost to follow-up.

**Figure 2:**
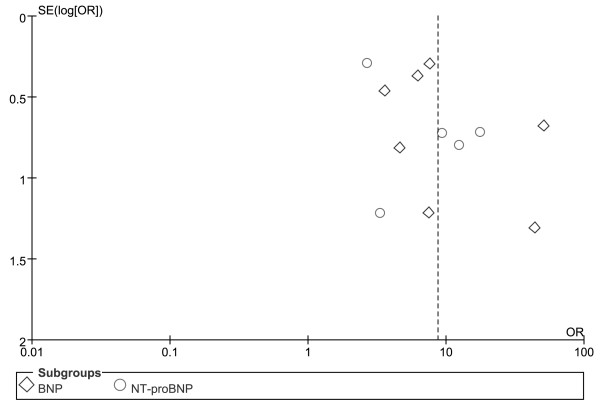
**Funnel plot for the predictive value of elevated BNP or NT-proBNP for mortality in patients with sepsis**. BNP = brain natriuretic peptide, NT-proBNP = N-terminal pro-B-type natriuretic peptide.

Regarding the predictive value of elevated BNPs for mortality, there was no evidence of significant publication bias by inspection of the funnel plot (Figure 2).

### Predictive value of BNPs on all-cause mortality

**Figure 3:**
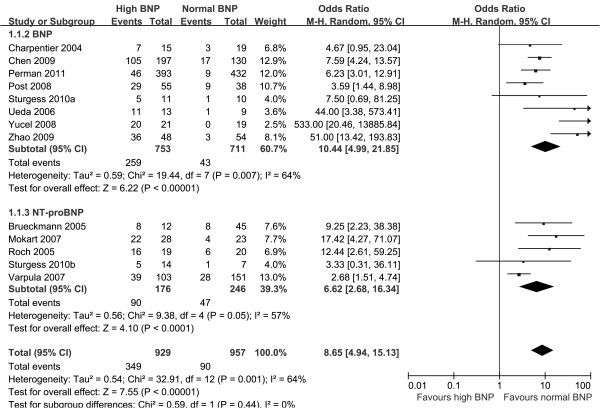
**Summary of odds ratio of mortality in septic patients with elevated BNP or NT-proBNP**. BNP = brain natriuretic peptide, NT-proBNP = N-terminal pro-B-type natriuretic peptide.

**Figure 4:**
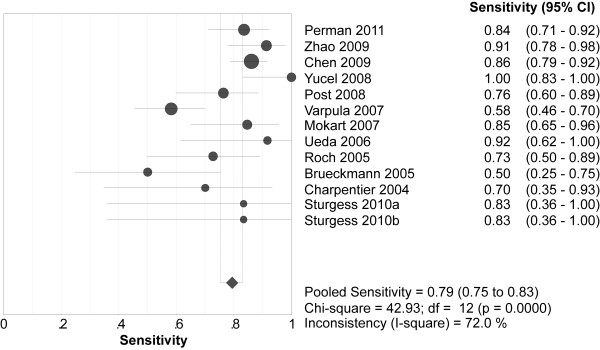
**Summary of sensitivity of elevated BNP or NT-proBNP in predicting mortality**. BNP = brain natriuretic peptide, NT-proBNP = N-terminal pro-B-type natriuretic peptide.

**Figure 5:**
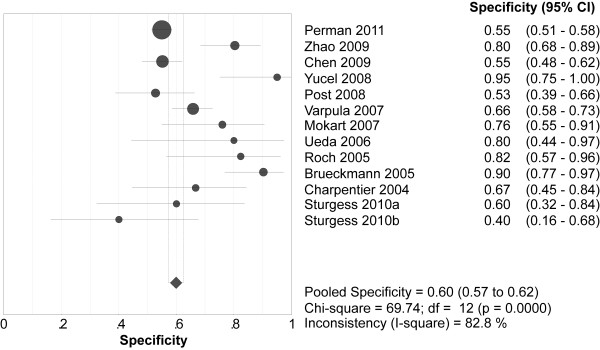
**Summary of specificity of elevated BNP or NT-proBNP in predicting mortality**. BNP = brain natriuretic peptide, NT-proBNP = N-terminal pro-B-type natriuretic peptide.

**Figure 6:**
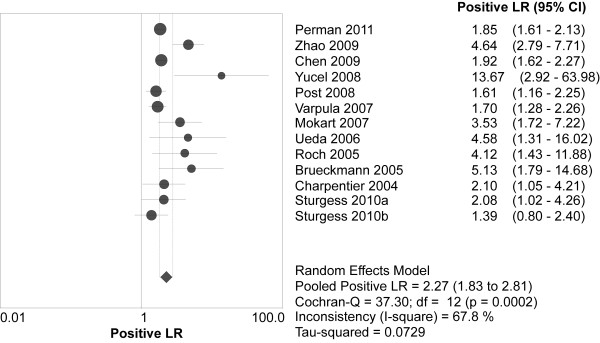
**Summary of the positive likelihood ratio of elevated BNP or NT-proBNP in predicting mortality**. BNP = brain natriuretic peptide, NT-proBNP = N-terminal pro-B-type natriuretic peptide.

**Figure 7:**
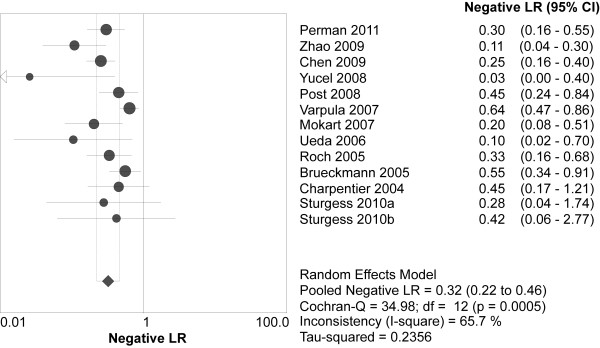
**Summary of the negative likelihood ratio of elevated BNP or NT-proBNP in predicting mortality**. BNP = brain natriuretic peptide, NT-proBNP = N-terminal pro-B-type natriuretic peptide.

Overall, 349 (37.6%) of the 929 patients with elevated BNPs died *vs *90 (9.4%) of the 957 patients with normal BNPs. Spearman's correlation coefficient between sensitivity and specificity was -0.132 (*P *= 0.667), suggesting no evidence of a threshold effect. Therefore, pooled estimates were calculated. Elevated BNPs were associated with a significantly increased risk of all-cause mortality (OR 8.65, 95% confidence interval (CI) 4.94 to 15.13, *P *< 0.00001, Figure 3) with significant heterogeneity (I^2 ^= 64%, *P *= 0.001). The pooled sensitivity and specificity were 79% (95% CI 75 to 83, Figure 4) and 60% (95% CI 57 to 62, Figure 5), respectively. The summary positive and negative likelihood ratios were 2.27 (95% CI 1.83 to 2.81, Figure 6) and 0.32 (95% CI 0.22 to 0.46, Figure 7), respectively.

The prior sensitivity analysis suggested that the heterogeneity was accounted for by two studies which had the minimum [28] and maximum [26] areas under the ROC curve. With the two studies excluded, the pooled OR was not significantly altered (OR 8.67, 95% CI 5.46 to 13.77, *P *< 0.00001) with no significant heterogeneity (I^2 ^= 33%, *P *= 0.13). Subgroup analyses suggested that the association between elevated BNPs and increased risk of all-cause mortality was consistent for BNP (OR 10.44, 95% CI 4.99 to 21.58, *P *< 0.00001; I^2 ^= 64%) [22-27,30,33] and NT-proBNP (OR 6.62, 95% CI 2.68 to 16.34, *P *< 0.0001; I^2 ^= 57%) [23,28,29,31,32], as well as blinding (OR 3.83, 95% CI 2.52 to 5.82, *P *< 0.00001; I^2 ^= 0%) [22,28,30,33] and no blinding (OR 14.24, 95% CI 6.56 to 30.93, *P *< 0.00001; I^2 ^= 63%) [23-27,29,31,32] of BNPs measurement to the outcome. According to our priori hypothesis, meta-regression analyses showed that patient population (*P *= 0.76), the underlying diseases (*P *= 0.12), optimal timing of BNPs measurement (*P *= 0.89), and mortality (*P *= 0.10) did not explain the demonstrated heterogeneity, respectively.

## Discussion

The present meta-analysis showed that elevated BNPs were associated with a significantly increased risk of mortality in patients with sepsis. The finding is consistent for both BNP and NT-proBNP. As such, measurement of BNPs may be a simple method of risk stratification in septic patients.

BNPs elevation in patients with sepsis can be considerably high, even though cardiac depression is not obvious. A retrospective study [34] suggested that BNPs level in septic patients with preserved systolic left ventricular function could be as high as that in patients admitted to the hospital with congestive heart failure because of severely impaired systolic left ventricular function. Aside from neurohormonal activation, several mechanisms are likely to account for increased BNPs levels in sepsis, including sepsis-induced biventricular dilatation [35], the stimulation of lipopolysaccharide [36] or proinflammatory cytokines [37,38], volume resuscitation [39] and sepsis-associated acute lung injury or acute respiratory distress syndrome [40]. Accordingly, elevated BNPs level in the presence of sepsis does not essentially mean cardiac dysfunction due to low specificity, while normal BNPs level could be used to rule out the need for further cardiac investigation, unless there are other clinical grounds that strongly suspect a significant cardiac disorder.

Clinical severity scores such as acute physiology and chronic health evaluation (APACHE) II and sequential organ failure assessment (SOFA) scores have been validated for mortality risk stratification, but are unwieldy and tend to be used more for audit and research than clinical decision making in sepsis. A rapidly available biochemical test that provides similar or better prognostic information could therefore be useful, e.g. to help discussions about prognosis with patients' relatives and decisions regarding earlier interventions. Several studies [25,41,42] showed that BNPs level was related to APACHE II and SOFA scores. In the present meta-analysis, two [25,27] of the included studies directly compared the prognostic value of BNPs measurement *vs *clinical severity scores and both suggested a better prognostic value of elevated BNPs in predicting mortality. Chen et al [25] reported a greater area under the ROC curve for the plasma BNP level than for APACHE Ⅱ score (0.737 *vs *0.664). Post et al [27] reported that regarding predicting 30-day mortality, the area under the ROC curve was greater for BNP than that for APACHE Ⅱ score and SOFA score (0.648 *vs *0.494 and 0.493, respectively). Considering the low sensitivity and high specificity of clinical severity scores, they can be combined with BNPs for aggregate analysis on the septic prognosis. What additional value of BNP brings to severity scores on sepsis risk stratification remains to be determined in further studies.

As a risk stratification tool, BNPs measurement is simple, inexpensive, reproducible, non-invasive, and widely available. These attributes make it highly suitable for serial testing to monitoring outcome risk over time, which may be superior to a single value of BNPs assessed on admission, because dynamic changes in BNPs concentration may reflect the development of sepsis. Furthermore, rather than necessarily dichotomizing risk as high or low, BNPs express risk as a continuum and this may be advantageous.

To date, several biomarkers have been identified to have some prognostic value in the field of sepsis. According to a prospective multicenter observational study [43] procalcitonin > 0.85 ng/ml was associated with an increased risk of death in a Cox regression analysis (hazard ratio 2.31, 95% CI 1.32-4.05, P = 0.003). In addition, both elevated cardiac troponin-T [44] and -I [45] were reported to be associated with higher mortality in septic patients. In the present study, pooled sensitivity and specificity of elevated BNPs for predicting mortality in septic patients were 79% and 60%, respectively. So far, none of the proposed prognostic markers had sufficient (more than 90%) sensitivity and specificity to predict which patients were at greater risk of dying due to sepsis [46]. Accordingly, a BNPs measurement may provide a better prognostic value in combination with other biomarkers, each mirroring different pathophysiological aspects. Further study was warranted to verify this hypothesis and evaluate the cost-effectiveness in sepsis.

In the past decade, several fully automated, rapid assays for determination of BNPs have become commercially available, including both high-throughput automated platforms and point of care tests. Although these existing BNPs assays correlate closely, BNPs assays are not currently analytical equivalent due to the lack of assay standardization [47]. Recently, a multicenter study conducted in 90 Italian laboratories demonstrated that there were significant differences in analytical characteristics and measured values among the most popular commercial methods for BNP and NT-proBNP [48]. Therefore, clinicians should be very careful when comparing results obtained by laboratories that use different methods.

An important concern of using BNPs in septic patients is the impact of renal insufficiency on these tests. Acute renal insufficiency occurs in 11 - 16% of critically ill patients who presented with sepsis [49,50]. A study by Goei et al [51] suggested that NT-proBNP had more favorable discriminative value in patients with a glomerular filtration rate more than 90 mL/min/1.73 m^2^, while it lost its prognostic value in patients with a glomerular filtration rate less than 30 mL/min/1.73 m^2^. No guidance on how BNPs values are adjusted for renal dysfunction is available today. Given the high prevalence and the impact of renal impairment on the BNPs values, it is preferable to adopt different cut-offs stratified by renal function in patients with sepsis.

There were several limitations in the present study. First, marked heterogeneity existed across the included studies in terms of population characteristics, BNPs assays, optimal cut-off point, follow-up period and definitions of endpoints. However, all the individual ORs favored the prognostic value of elevated BNPs in predicting mortality, indicating that the heterogeneity was entirely quantitative. Although there was uncertainty regarding the strength of the association, current evidence suggested that there was a significant correlation between an elevated BNPs level and an increased risk of mortality in septic patients. Second, the optimal timing of BNPs measurement varied across the studies, including the day on admission, and day 2 and day 5 after admission. It can be partly accounted for by the difficulty in determining the time of onset of sepsis and, hence, the time of patient recruitment. Third, it should be noted that the pooled estimates reflected unadjusted associations between BNPs and all-cause mortality. Five [22,25,27,29,31] of the included studies provided adjusted ORs accounting for confounders. Pooled analysis of the adjusted ORs also suggested that elevated BNPs were correlated with a significantly increased risk of mortality in septic patients (random-effects model, OR 1.87, 95% CI 1.30 to 2.69, *P *= 0.001). Because the ORs in the five studies were adjusted by different confounders with different regression models and the data reported on adjusted ORs were limited, we thus adopted unadjusted mortality data for pooled analyses in the present study. Finally, we could not determine the ideal cutoff points for BNP and NT-proBNP tests because we did not have the raw data to map out ROC curves. To determine whether there is a single threshold or a few important BNP or NT-proBNP thresholds (e.g., age dependent), further evaluation in prespecified groups of larger numbers of patients is needed.

## Conclusions

This systematic review and meta-analysis suggests that an elevated BNP or NT-proBNP level may prove to be a powerful predictor of mortality in patients with sepsis. This test appears to represent a rapid and relatively inexpensive method to enhance mortality prediction in sepsis. Future larger and more adequately powered prospective studies are warranted to clarify the assay standardization, the optimal cut-off, and the prognostic value of BNPs in conjunction with other biomarkers.

## Key messages

• The literature shows that an elevated BNP or NT-proBNP level is a powerful predictor of mortality in patients with sepsis.

• This test appears to represent a rapid and relatively inexpensive method to enhance mortality prediction in sepsis.

• Larger adequately powered prospective studies are warranted to clarify the assay standardization, the optimal cut-off, and the prognostic value of BNPs in conjunction with other biomarkers in future.

## Abbreviations

BNP: brain natriuretic peptide; NT-proBNP: N-terminal pro-B-type natriuretic peptide; ICU: intensive care unit; OR: odds ratio; CI: confidence interval; ROC: receiver operating characteristic; APACHE: acute physiology and chronic health evaluation; SOFA: sequential organ failure assessment.

## Competing interests

The authors declare that they have no competing interests.

## Authors' contributions

Study concept and design: FW, YW, LT, JL and XD. Acquisition of data: FW, YW and LT. Analysis and interpretation of data: WZ, FC, LB and TX. Drafting of the manuscript: FW, YW and LT. Critical revision of the manuscript for important intellectual content: JL and XD. Statistical analysis: FW and YW. Administrative, technical, and material support: WZ, FC, LB and TX. Study supervision: JL and XD. All authors have read and approved the manuscript for publication.

## Supplementary Material

Additional file 1 **The Original Quality Assessment of Diagnostic Accuracy Studies Checklist**. Click here for file
