# Supplementary material for: Brain natriuretic peptide for prediction of mortality in patients with sepsis: a systematic review and meta-analysis
Source: Crit Care. 2012 May 6;16(3):R74. doi: 10.1186/cc11331 (PMC3580616; doi:10.1186/cc11331)
Supplement: Additional file 1 — The Original Quality Assessment of Diagnostic Accuracy Studies Checklist. [file cc11331-S1.DOC]

**The Original Quality Assessment of Diagnostic Accuracy Studies Checklist** [1]:

1. Was the spectrum of patients representative of the patients who will receive the test in practice?

2. Were selection criteria clearly described?

3. Is the reference standard likely to correctly classify the target condition?

4. Is the time period between reference standard and index test short enough to be reasonably sure that the target condition did not change between the two tests?

5. Did the whole sample or a random selection of the sample, receive verification using a reference standard of diagnosis?

6. Did patients receive the same reference standard regardless of the index test result?

7. Was the reference standard independent of the index test (i.e. the index test did not form part of the reference standard)?

8. Was the execution of the index test described in sufficient detail to permit replication of the test?

9. Was the execution of the reference standard described in sufficient detail to permit its replication?

10. Were the index test results interpreted without knowledge of the results of the reference standard?

11. Were the reference standard results interpreted without knowledge of the results of the index test?

12. Were the same clinical data available when test results were interpreted as would be available when the test is used in practice?

13. Were uninterpretable/ intermediate test results reported?

14. Were withdrawals from the study explained?

Given that the eligible studies were of a prognostic nature, the included studies were assessed for methodological and reporting quality according to the Quality Assessment of Diagnostic Accuracy Studies checklist [1]. Accordingly, the formulation “index test”, “target condition”, and “reference standard” were replaced by “BNPs concentrations”, “all-cause mortality”, and “outcome”, respectively. Moreover, criteria 3, 4, 7 and 13 of the original checklist [1] were considered as not applicable in this context. Criterion 9 (execution of outcome assessment) of the original checklist were regarded as not applicable for the studies addressing ICU or in-hospital all-cause mortality only.

**Modified Checklist Used in the Present Study**:

1. Was the spectrum of patients representative of the patients who will receive the natriuretic peptide concentration measurement in practice?

2. Were selection criteria clearly described?

5. Did the whole sample or a random selection of the sample, receive verification using a outcome assessment?

6. Did patients receive the same outcome assessment regardless of the natriuretic peptide concentration measurement result?

8. Was the execution of the natriuretic peptide concentration measurement described in sufficient detail to permit replication of the test?

10. Were the natriuretic peptide concentration measurement results interpreted without knowledge of the results of the outcome assessment?

11. Were the outcome assessment results interpreted without knowledge of the results of the natriuretic peptide concentration measurement?

12. Were the same clinical data available when test results were interpreted as would be available when the natriuretic peptide concentration measurement t is used in practice?

14. Were withdrawals from the study explained?

**Reference**:

1. Whiting P, Rutjes AW, Reitsma JB, Bossuyt PM, Kleijnen J: **The development of quadas: A tool for the quality assessment of studies of diagnostic accuracy included in systematic reviews.** *BMC Med Res Methodol* 2003, 3: 25.
